# Isolation and proteomic study of fish liver lipid droplets

**DOI:** 10.52601/bpr.2023.230004

**Published:** 2023-06-30

**Authors:** Yuwei Sun, Jian Heng, Feng Liu, Shuyan Zhang, Pingsheng Liu

**Affiliations:** 1 National Laboratory of Biomacromolecules, CAS Center for Excellence in Biomacromolecules, Institute of Biophysics, Chinese Academy of Sciences, Beijing 100101, China; 2 State Key Laboratory of Membrane Biology, Institute of Zoology, Chinese Academy of Sciences, Beijing 100101, China; 3 Institute of Stem Cell and Regeneration, Chinese Academy of Sciences, Beijing 100101, China; 4 University of Chinese Academy of Sciences, Beijing 100049, China

**Keywords:** Lipid droplet, Fish, Liver, Proteomics, Plin2

## Abstract

Lipid droplets (LDs) are a neutral lipid storage organelle that is conserved in almost all species. Excessive storage of neutral lipids in LDs is directly associated with many metabolic syndromes. Zebrafish is a better model animal for the study of LD biology due to its transparent embryonic stage compared to other organisms. However, the study of LDs in fish has been difficult due to the lack of specific LD marker proteins and the limitation of purification technology. In this paper, the purification and proteomic analysis of liver LDs of fish including zebrafish and *Carassius auratus* were performed for the first time. 259 and 267 proteins were identified respectively. Besides most of the identified proteins were reported in previous LD proteomes of mammals, indicating the similarity between mammal and fish LDs. We also identified many unique proteins of liver LDs in fish that are involved in the regulation of LD dynamics. Through morphological and biochemical analysis, we found that the marker protein Plin2 of zebrafish LD was located on LDs in Huh7 cells. These results will facilitate further study of LDs in fish and liver metabolic diseases using fish as a model animal.

## INTRODUCTION

LD is an intracellular organelle consisting of a neutral lipid core surrounded by a phospholipid monolayer membrane coated with various types of proteins (Farese and Walther 2009; Fujimoto *et al.* 2008; Martin and Parton 2006; Murphy 2012; Thiam *et al*. 2013). The dynamics of LDs is closely related to many metabolic diseases, such as obesity, fatty liver, type 2 diabetes mellitus, and atherosclerosis (Krahmer *et al.* 2013a; Maeda *et al.* 2005; Xu *et al*. 2018). PAT/PLIN family proteins, such as perilipin/PLIN1, ADRP/PLIN2, and Tip47/PLIN3, as useful marker proteins, have facilitated the purification of LDs, which provides the detailed molecular composition of proteins and lipids of the organelle (Sztalryd and Brasaemle 2017). With these molecular compositions, plus related functional studies, the understanding of LDs has developed significantly in the last two decades (Zhang and Liu 2019). LDs have been found to interact with other cellular organelles including the cytoskeleton, endoplasmic reticulum (ER), endosomes, mitochondria, and peroxisomes (Goodman 2008; Murphy *et al.* 2009; Zehmer *et al.* 2009; Zhang *et al*. 2010) and be involved in several processes including lipid metabolism, cell signal transduction, protein storage and degradation, membrane trafficking, and nucleic acid handling (Cermelli *et al.* 2006; Ducharme and Bickel 2008; Fujimoto and Parton 2011; Goodman 2009; Harris *et al*. 2011; Zimmermann *et al*. 2004; Yu *et al.* 1998, 2000).

For more than 200 years, fish have been used as a model organism such as zebrafish and *Carassius auratus* (Ribas *et al*. 2014). Zebrafish have been used typically in biomedical research in recent years, which is suitable to investigate the mechanism and process associated with metabolic diseases, including diet-induced obesity, type 2 diabetes mellitus, atherosclerosis and liver-related diseases in humans (Teame *et al.* 2019). Drugs for alleviating metabolic syndromes in humans have also been approved effectively in the zebrafish model (Asaoka *et al.* 2013; Misselbeck *et al.* 2019; Nakayama *et al.* 2018, 2020). One reason is that zebrafish embryo is transparent and develops rapidly outside of the uterus. Another reason is zebrafish have been fully sequenced to a very high quality. It is shown that humans and zebrafish share 70% of the same genes and 84% of human genes known to be associated with human diseases have a counterpart in zebrafish (Ribas *et al*. 2014). Similarly, *Carassius auratus* which is close to the zebrafish in evolution is a freshwater fish species that is widely distributed in China for its fast growth as well as high nutritional and economic value (Blanco *et al*. 2022). *Carassius auratus* is the oldest model species with the similar organs and tissues as mammalian and is also been primarily used in applied studies of human health and diseases especially those pertaining to neurology and vision as well as many organs or biological processes, including aquatic toxicology and gut motility (Blanco *et al*. 2022; Mensah *et al.* 2018).

LDs have been found in the cell of almost all organisms from bacteria to mammals (Murphy 2001; Yang *et al*. 2012; Zhang and Liu 2017) and the studies of various organisms including bacteria, yeast, plant, insect, *C. elegans*, and mammals are facilitated by the development of proteomic technology, which provides useful information about composition and function of cellular LD proteins (Bartz *et al.* 2007; Beilstein *et al.* 2013; Beller *et al.* 2006; Bouchoux *et al.* 2011; Brasaemle *et al.* 2004; Cermelli *et al.* 2006; Crunk *et al.* 2013; D'Aquila *et al.* 2015; Ding *et al.* 2012a, b; Eichmann *et al.* 2015; Fujimoto *et al.* 2004; Grillitsch *et al.* 2011; Khor *et al.* 2014; Kim *et al.* 2006; Krahmer *et al.* 2013b; Liu *et al*. 2004; Na *et al.* 2015; Park *et al.* 2004; Pereira *et al.* 2013; Saka *et al.* 2015; Sato *et al.* 2006; Shi *et al.* 2013; Su *et al.* 2014; Turro *et al.* 2006; Umlauf *et al.* 2004; Vrablik *et al.* 2015; Yu *et al.* 2015; Zhang *et al*. 2011; Zhang *et al.* 2012). While the proteomic methods are rarely used to study the LD proteome of fish, the only study is the research on LD-associated proteins of adipose tissue in grass carp and due to the limitation of LD purification techniques, the marker protein of fish liver LD has not been identified through biochemical method (Tian *et al.* 2020). Besides, the LD proteins of fish liver have not been studied. Fish is an excellent research model for studying many liver diseases since its liver there is highly homologous to mammal liver (Chen *et al.* 2018; Goessling and Sadler 2015). Therefore, the study of fish liver LD proteins is essential to further the understanding of the role of LDs and contribute to the study of liver pathophysiology.

In this study, we modified the method for purifying LDs from zebrafish and *Carassius auratus* liver and performed a proteomic study to identify LD proteins in liver fish, to explore the LD function. Proteomic analysis of zebrafish liver LDs identified 259 proteins, and 87 proteins were newly identified in our study. Proteomic analysis of *Carassius auratus* liver LDs identified 267 proteins, and 133 proteins were newly identified in our study. We analyzed the LD proteins based on their cellular functions and subcellular locations, and the zebrafish Plin2 of the PAT family was also identified as localized on LD of mammalian liver cells as marker proteins.

## RESULTS

### Isolation of LDs from zebrafish liver

**Figure 1:**
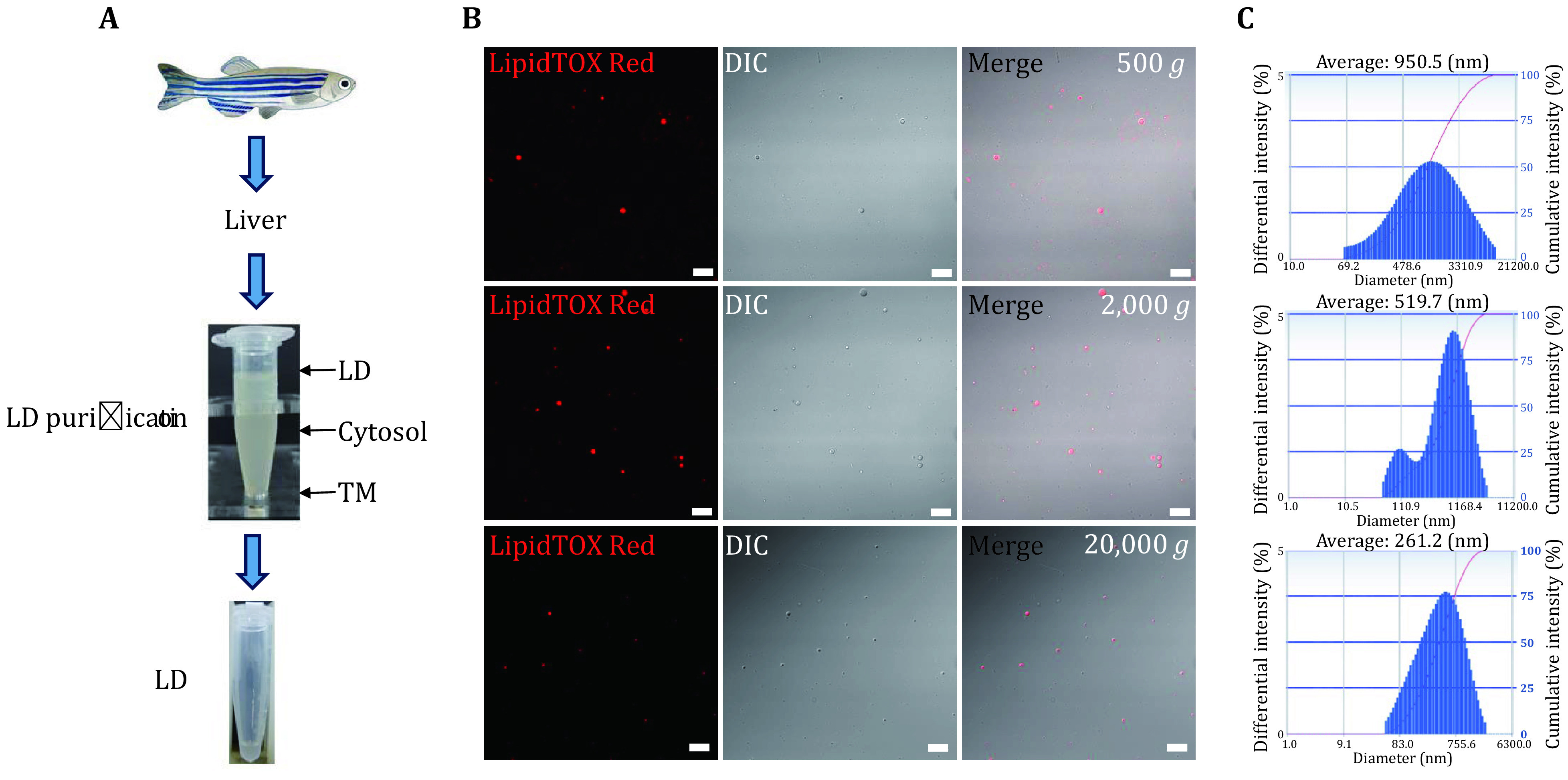
Isolation of LDs from zebrafish liver. **A** Flowchart of isolation of LDs from zebrafish liver. Livers of adult zebrafishes were collected and homogenized, and the fraction of PNS was collected and subjected to centrifuge at 500 *g*, 2,000 *g* and 20,000 *g*. After centrifugation, the white layer on the top of the gradient was collected as LD fraction, the pellet at the bottom was TM, and the middle solution was collected as Cyto. The LD fraction was then washed three times and kept in an Eppendorf tube for further usage. **B** Isolated LDs were first analyzed by confocal microscopy with LipidTOX Red staining, DIC imaging, and merged images. Bar = 10 μm. **C** The size of isolated LDs was measured by a Delsa Nano C particle analyzer

To understand LD dynamics in fish, the organelle was isolated from a model fish, zebrafish, by a modified method described before (Fig. 1A) (Ding *et al.* 2013). We centrifuged the PNS at 500 *g*, 2,000 *g*, and 20,000 *g* to isolate LDs with different sizes. To further verify the quality of isolated LDs, LDs were analyzed by confocal microscopy after LipidTOX Red staining, with DIC imaging, and merged images. The results showed that the isolated LDs appeared as spheres in DIC images that overlapped with LipidTOX Red signals. Other membrane contaminations were hardly observed, indicating a high purity of isolated LDs (Fig. 1B). Subsequently, the size of isolated LDs was measured by a Delsa Nano C particle analyzer. The results showed that the average diameter of isolated LDs from 500 *g* centrifugation was 950.5 nm, LDs from 2,000 *g* was 519.7 nm, and LDs from 20,000 *g* was 261.2 nm (Fig. 1C). These results showed the higher the centrifugation speed, the smaller the LDs, which is consistent with previous studies (Ding *et al.* 2013).

**Figure 2:**
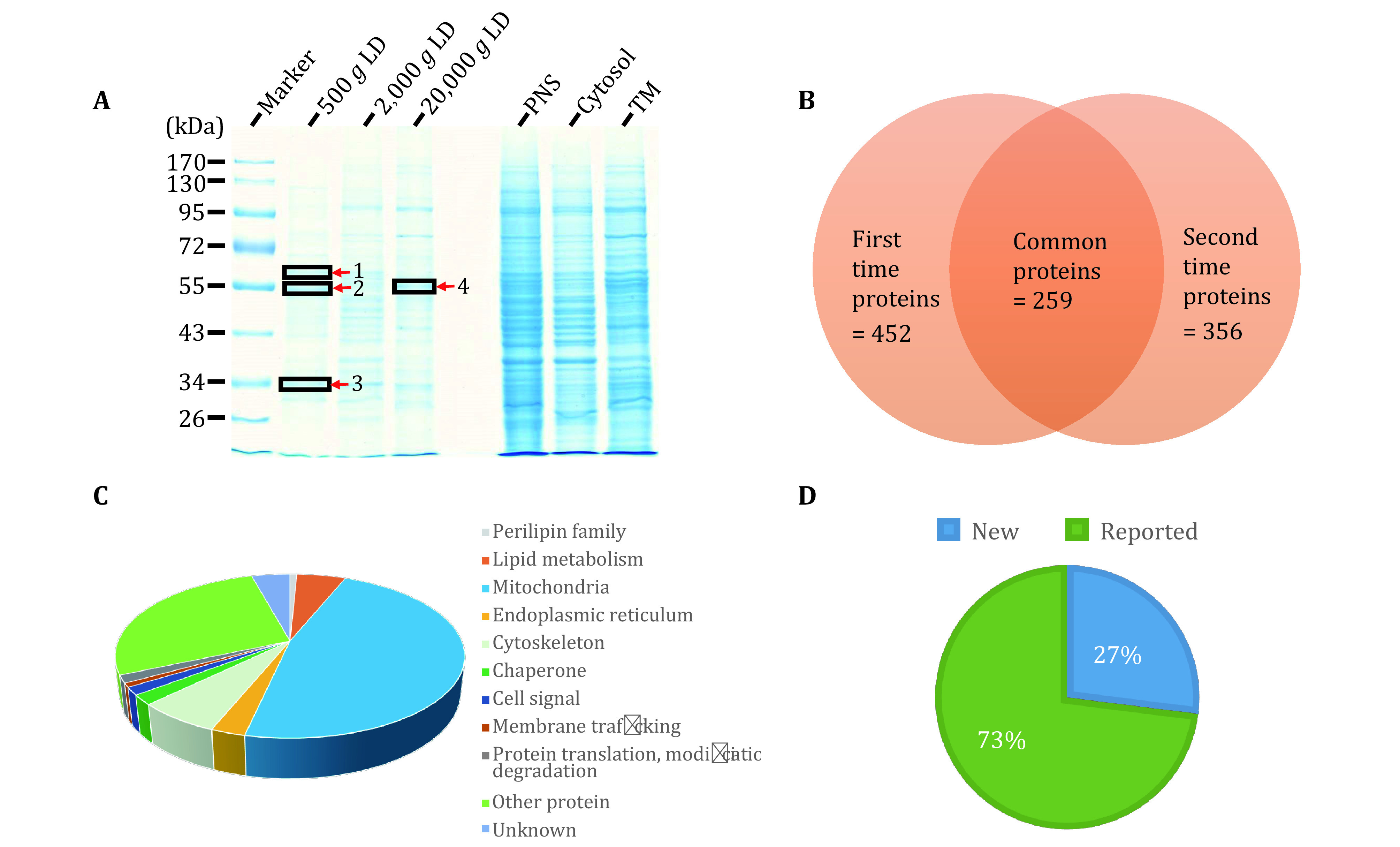
Proteomic analysis of isolated LDs from zebrafish liver. **A** Proteins were separated by SDS-PAGE after extracting from LD, TM, Cyto, and PNS fractions, and then stained by colloidal blue. Red arrowheads marked unique protein bands of LDs. **B** Proteins of LDs were precipitated by acetone and then subjected to nano-LC-ESI-LTQ Orbitrap XL MS/MS analysis. The Venn diagram showed the overlap of two independent mass spectrum results. **C** The 259 proteins of LDs identified in two samples were categorized into 11 groups: perilipin family, lipid metabolism, mitochondria, endoplasmic reticulum, cytoskeleton, chaperone, cell signal, membrane trafficking, protein translation and modification degradation, other protein and unknown function protein. **D** Proteins of LDs of two independent mass spectrum results were categorized into previously reported proteins and newly identified proteins in this study

The quality of isolated LDs was further examined using gel electrophoresis to analyze the LD protein profile and determine the unique proteins. Briefly, equal amounts of proteins from LD, PNS, Cyto, and TM fractions were separated by SDS-PAGE, and stained using colloidal blue. The stained gel presents that the protein pattern of isolated LDs was significantly different from the other three cellular fractions, also indicating a high quality of isolated LDs (Fig. 2A).

### Proteomic analyses of isolated LDs from zebrafish liver

The examinations of morphology and biochemistry verified the high quality of isolated LDs, demonstrating that the isolated LDs were suitable for proteomic analysis. Thus, the LD proteins were subjected to proteomic analysis using nano-LC-ESILTQ Orbitrap XL MS/MS. To improve the reliability of this proteomic analysis, two biological samples were analyzed and two independent proteomes of LDs were obtained. Among the two independent LD proteomic studies, 259 proteins were identified in both analyses with at least two unique peptides (supplementary Table S1 and Fig. 2B). Among the identified proteins, 73% (190 of 259) proteins had been identified in previous LD proteomic studies, which indicates the reliability of the isolation of LDs and proteomic procedure, and the remaining 27% (69 of 259) proteins were newly identified (Fig. 2D).

The 259 proteins were categorized into 11 groups based on their cellular functions and subcellular locations using the UniProt database. As shown in the supplementary Table S1, two perilipin family proteins Plin2 and Plin3 were identified. The most abundant proteins identified were mitochondrial proteins (47%; 123 proteins), and ER proteins constituted 3% (7 proteins), suggesting that LDs dynamically interact with other cellular organelles, especially with mitochondria, which was also observed in other LD proteomic studies (Jagerstrom *et al.* 2009; Ohsaki *et al.* 2008; Ozeki *et al.* 2005; Su *et al.* 2014; Zehmer *et al.* 2009; Zhang *et al.* 2012). Furthermore, 14 proteins (5%) were found to be involved in lipid metabolism, indicating that LDs in fish liver play a role in maintaining the cellular lipid homeostasis (Walther and Farese 2012). 17 cytoskeleton proteins were identified, suggesting a possibility of LD movement (Welte *et al.* 1998). In addition, chaperone, cell signal, protein translation and modification, protein degradation, and membrane trafficking proteins constituted 2% (5 proteins), 2% (4 proteins), 2% (4 proteins), and 1% (2 proteins), respectively. Meanwhile, other functional proteins (27%; 71 proteins) and unknown functional proteins (4%; 11 proteins) were identified (Fig. 2C).

To identify abundant proteins, five slices were cut from the SDS-PAGE marked by arrowheads in Fig. 2A and supplementary Fig. S2, and subjected to proteomic analysis. Supplementary Table S2 represents the abundant proteins of each band. Acyl-CoA synthetase and protein disulfide-isomerase were found in band 1 close to 63 kDa. ATP synthase, Plin2 and Plin3 were identified in band 2 close to 55 kDa. Short-chain dehydrogenase/reductase family 16C and ribosomal protein S3 were detected in band 3 close to 34 kDa. DnaJ (Hsp40) and protein disulfide-isomerase were found in band 4 close to 56 kDa. Besides ATP synthase, Plin2 and Plin3 were also identified in band 5 at almost 55 kDa, which is consistent with band 2 (supplementary Table S2 and Fig. S2).

**Figure 3:**
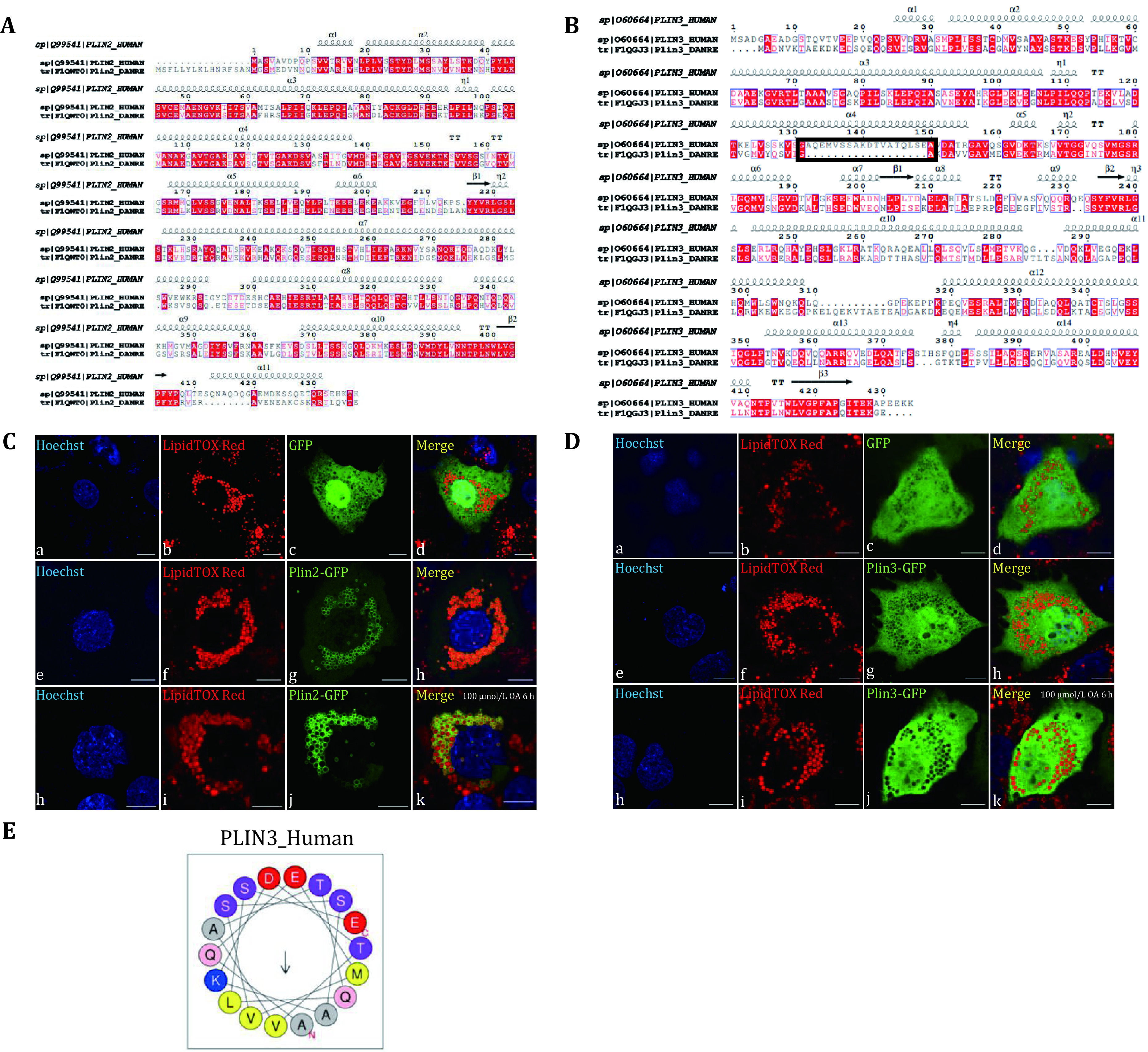
Localization of Plin2 and Plin3 of zebrafish in Huh7 cells. **A** The amino acid sequence alignment of Plin2 of human and zebrafish. The amino acid sequences were compared by Clustal X and visualized by ESPript3.0. **B** The amino acid sequence alignment of Plin3 of human and zebrafish. The amino acid sequences were compared by Clustal X and visualized by ESPript3.0. The black frame showed the absence of sequence in zebrafish Plin3. **C** Zebrafish *plin2* was cloned into GFP plasmid and transiently overexpressed in Huh7 cells. The cells were imaged by an Olympus FV1200 confocal microscope. Upper panels: the vector GFP overexpressed in Huh7 cells; Middle panels: Plin2-GFP overexpressed in Huh7 cells; Lower panels: Plin2-GFP overexpressed in Huh7 cells and treated with 100 μmol/L OA for 6 h. Green: GFP; Red: LipidTOX Red; Blue: Hoechst. Bar = 10 μm. **D** Zebrafish *plin3* was cloned into GFP plasmid and transiently overexpressed in Huh7 cells. The cells were imaged by an Olympus FV1200 confocal microscope. Upper panels: the vector GFP overexpressed in Huh7 cells; Middle panels: Plin3-GFP overexpressed in Huh7 cells; Lower panels: Plin3-GFP overexpressed in Huh7 cells and treated with 100 μmol/L OA for 6 h. Green: GFP; Red: LipidTOX Red; Blue: Hoechst. Bar = 10 μm. **E** Helical wheel projection of sequences of human PLIN3 in the black frame was predicted by HeliQuest

After analyzing the proteomics of LDs from zebrafish liver, it was shown that the main proteins Plin2 and Plin3 belonging to the PLIN family were detected in almost all of the results of proteomics at the same time (Figs. 3A and 3B). The consistent identification of PLINs in main bands further verified the proper isolation of LDs from zebrafish liver.

### Localization of Plin2 and Plin3 of zebrafish in Huh7 cells

In order to further examine the reliability of the zebrafish proteomics, we verified the localization of Plin2 and Plin3. Due to the lack of a proper zebrafish cell line, *plin2* and *plin3* were cloned into the GFP plasmid and then transfected into the human liver cell line Huh7 to verify the localization.

It could be seen that the green Plin2-GFP fusion protein formed circle structures around the LDs stained by LipidTOX Red, indicating that Plin2 in zebrafish is indeed localized on the LDs, which proved the reliability of the isolation of LDs and proteomics of zebrafish liver (Fig. 3C). However, it was found that the Plin3-GFP fusion protein was not localized in LDs like Plin3 in other mammalian cells. It was localized in cytosol and diffused throughout the cell even with OA treatment (Fig. 3D). Previous studies showed that the targeting mechanism of PLINs was highly conserved and the 11-mer repeat regions were sufficient for LD targeting through folding into amphipathic helices on the LD surface (Rowe *et al.* 2016). In order to explore the reason why the localization of Plin3 is cytosol, the alignment of Plin2 and Plin3 sequences of zebrafish and humans were further analyzed, respectively. The similarity of sequences for LD targeting between Plin2 of zebrafish and humans is high, and this may be the reason for the localization of zebrafish Plin2 on LDs, which is consistent with the targeting of human Plin2 (Fig. 3A). When Plin3 sequence of zebrafish was compared to Plin3 sequence of humans, it showed the similarity of sequences for LD targeting between Plin3 of zebrafish and humans is lower than Plin2, and more importantly, there was a deletion in the fourth α-helix region in black frame (Fig. 3B). The amphiphilic helical domain was predicted by Heliquest, which showed that the sequence of human Plin3 is amphipathic (Fig. 3E). Previous studies have identified that the minimal sequence of Plin3 necessary for complete recruitment to LDs was located between residues 87–209 (Bulankina *et al.* 2009). The missing sequence residues 132–149 in Plin3 of zebrafish were found in the 11-mer region responsible for LD localization in human. Therefore, these missing residues may be important for the localization of zebrafish Plin3 to human Huh7 cells.

### Isolation of LDs from *Carassius auratus* liver

**Figure 4:**
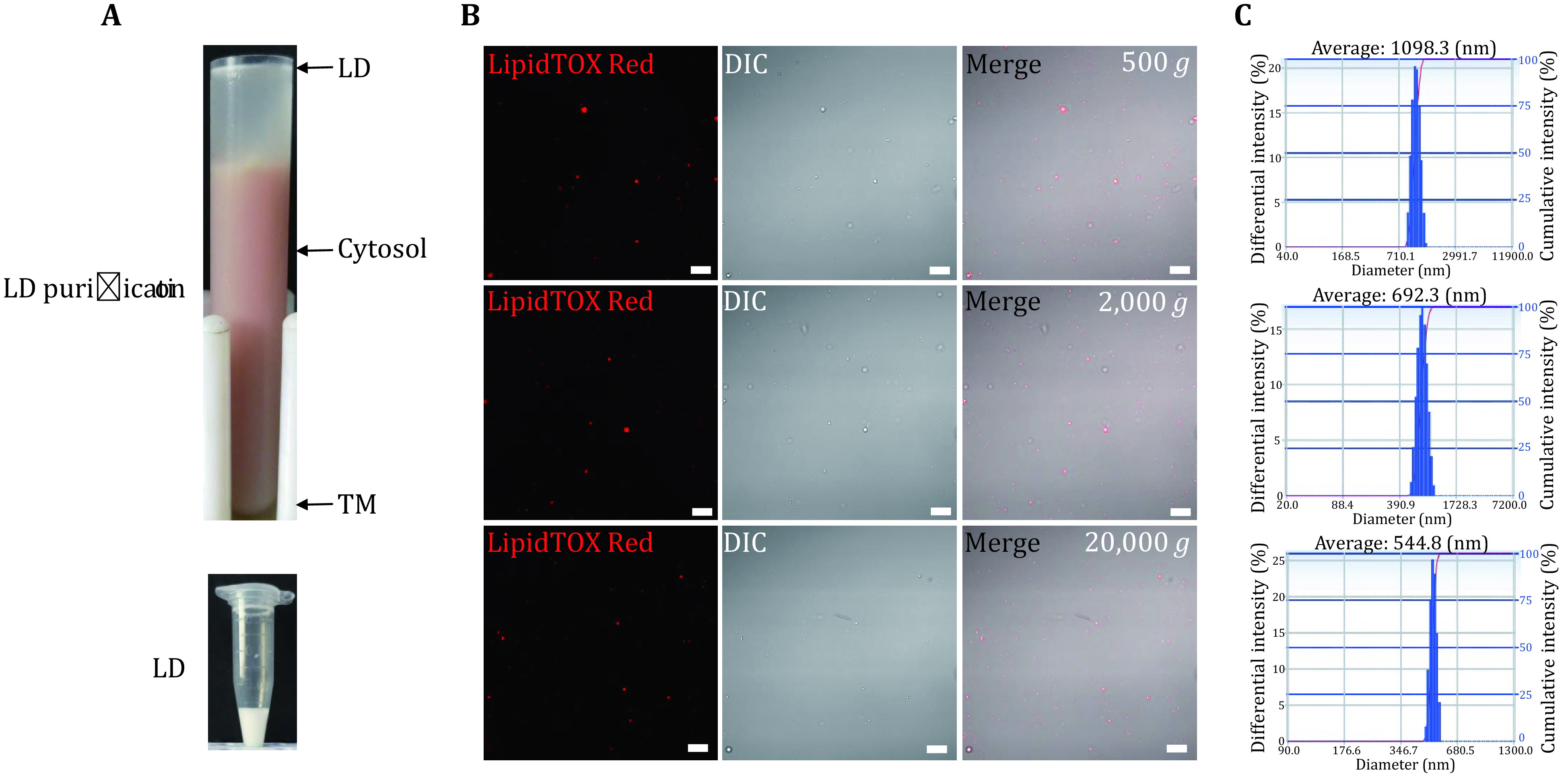
Isolation of LDs from *Carassius auratus* liver. **A** Livers of *Carassius auratus* were collected and homogenized by dounce, the fraction of PNS was collected after centrifugation at 500 *g* and subjected to separation of LD fraction. After centrifugation with the indicated speed, the white layer on the top of the gradient was collected as LD fraction, the pellet at the bottom was resuspended as TM, and the middle solution was collected as Cyto. The LD fraction was then washed three times and kept in an Eppendorf tube as isolated LDs for further usage. **B** Isolated LDs were first analyzed by confocal microscopy with LipidTOX Red staining, DIC imaging, and merged images. Bar = 10 μm. **C** The size of isolated LDs was measured by a Delsa Nano C particle analyzer

To extend our knowledge of fish liver LDs, *Carassius auratus* was selected and their livers were collected. A similar procedure for isolating LDs from zebrafish liver was applied to isolate LDs from *Carassius auratus* liver (Fig. 4A). Briefly, LDs with different sizes were isolated by centrifugation at 500 *g*, 2,000 *g*, and 20,000 *g*, respectively. To verify the quality of isolated LDs, they were analyzed by confocal microscopy with LipidTOX Red staining, DIC imaging, and merged images. The results showed that the isolated LDs appeared as spheres in DIC images that overlapped with LipidTOX Red staining signals. Similarly, other membrane contaminations were hardly observed, indicating a high purity of isolated LDs (Fig. 4B). Subsequently, the size of isolated LDs was also measured by a Delsa Nano C particle analyzer. The results showed that the average diameter of LDs isolated at 500 *g* was 1,098.3 nm, LDs from 2,000 *g* was 692.3 nm, and the 20,000 *g* isolated was 544.8 nm (Fig. 4C). These results were similar to the studies of zebrafish LDs above.

**Figure 5:**
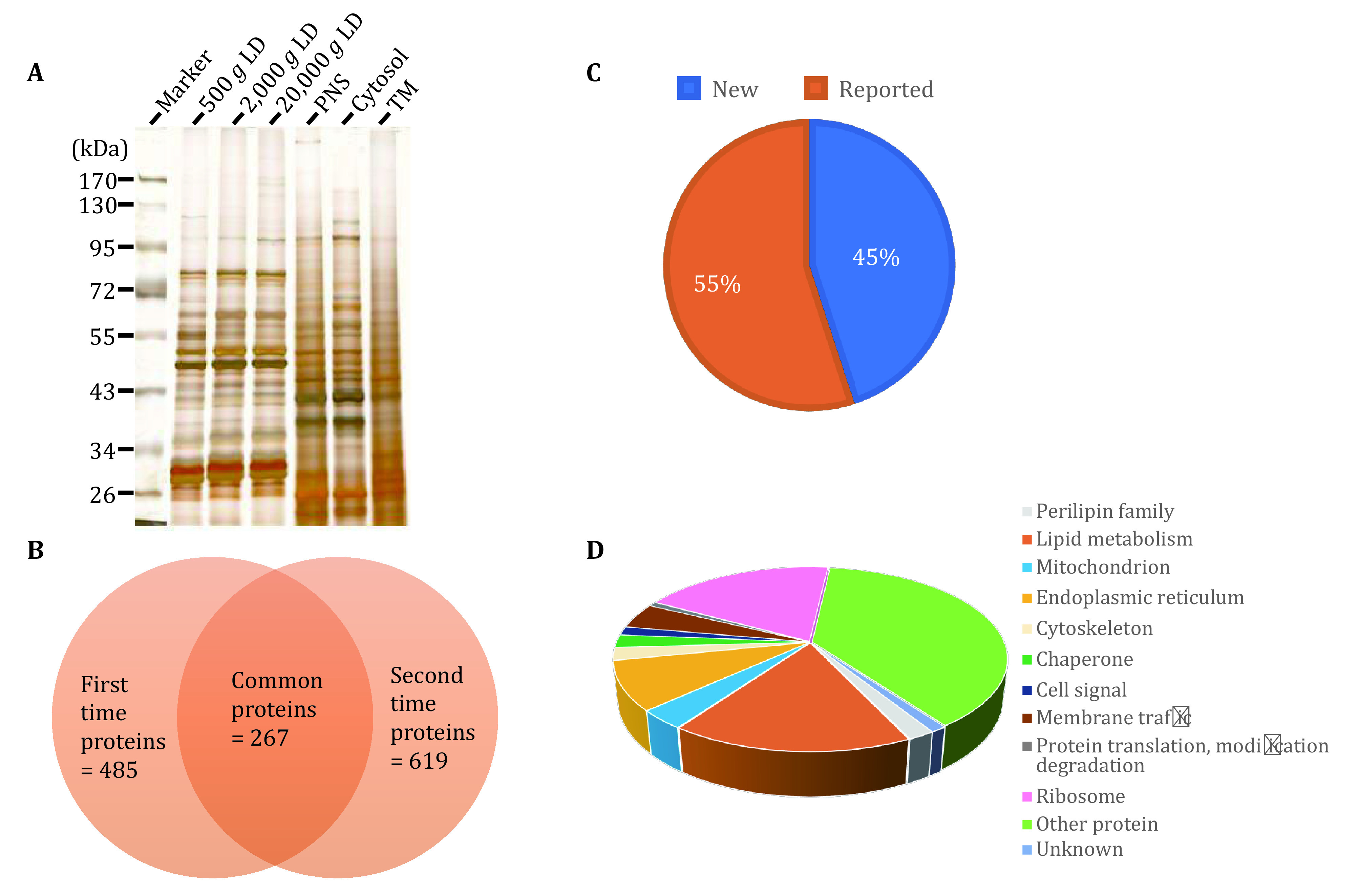
Proteomic analyses of isolated LDs from *Carassius auratus* liver. **A** Proteins were separated by SDS-PAGE after extracting from LD, TM, Cyto and PNS fractions, and stained by silver staining. **B** Proteins of LDs were precipitated by acetone and then subjected to nano-LC-ESI-LTQ Orbitrap XL MS/MS analysis. The Venn diagram showed the overlap of two independent mass spectrum results. **C** Proteins of LDs of two independent mass spectrum results were categorized into previously reported proteins and newly identified proteins in this study. **D** The 267 proteins of LDs identified in two samples were categorized into 12 groups: perilipin family, lipid metabolism, mitochondria, endoplasmic reticulum, cytoskeleton, chaperone, cell signal, membrane trafficking, protein translation and modification degradation, ribosome, other protein and unknown function protein

The quality of isolated LDs was further verified using gel electrophoresis to analyze the LD protein profile. Briefly, equal amounts of proteins from LD, PNS, Cyto, and TM fractions were separated by SDS-PAGE, and stained by silver staining. The stained gel presents that the protein pattern of isolated LDs was unique compared to the other three cellular fractions, also indicating a high quality of isolated LDs (Fig. 5A).

### Proteomic analyses of isolated LDs from *Carassius auratus* liver

The examinations of morphology and biochemistry verified the high quality of isolated LDs, demonstrating that the isolated LDs were suitable for proteomic analysis. In order to study the proteome of *Carassius auratus*, the LD proteins were subjected to the proteomic analysis using nano-LC-ESILTQ Orbitrap XL MS/MS. To improve the reliability of this proteomic analysis, two independent LD preparations were analyzed and two independent proteomes of LDs were obtained. From two independent LD proteomic studies, 267 proteins were identified in both analyses with at least two unique peptides (supplementary Table S3 and Fig. 5B). Slightly different from the zebrafish proteome, 55% (146 of 267) of the identified proteins had been reported in previous LD proteomic studies, which indicated a good reproducibility of the isolation of LDs and proteomic procedure, and the remaining 45% (121 of 267) proteins were newly identified (Fig. 5C).

The 267 proteins were categorized into 12 groups based on their functional characteristics and subcellular locations using the UniProt database. After analyzing the proteomics of LDs, three perilipin family proteins Plin1, Plin2 and Plin3 were identified. 45 proteins (17%) were found to be involved in lipid metabolism, indicating that LDs work in fish liver to maintain the cellular lipid homeostasis, and the ratio is increased obviously compared to that in LD proteomes of zebrafish liver. 50 proteins (19%) were ribosome proteins, which are components of the large ribosomal subunit responsible for the synthesis of proteins in the cell, and the ratio is increased obviously compared to that in LD proteomes of zebrafish liver. ER proteins (9%; 23 proteins) and mitochondrial proteins (3%, 9 proteins) were identified, suggesting that LDs dynamically interact with other cellular organelles, and the interaction was also suggested by LD proteomic studies of zebrafish. Furthermore, cytoskeleton, chaperone, cell signal, protein translation and modification, protein degradation, and membrane trafficking proteins constituted 2% (6 proteins), 2% (6 proteins), 2% (4 proteins), 1% (2 proteins), and 4% (12 proteins), respectively. In addition, other function proteins (38%; 102 proteins) and unknown function proteins (1%; 3 proteins) were identified (supplementary Table S3 and Fig. 5D). Overall, the proteomic results were similar to the proteomics of zebrafish, which further verified the proper isolation of LDs from *Carassius auratus* liver.

## DISCUSSION

Fish is a good food source as well as an excellent model organism for biological research. To understand lipid storage and metabolism of it, we carried out an isolation and proteomics studies of LDs from the livers of a model animal, zebrafish, and *Carassius auratus* with a modified method previously developed in our lab (Ding *et al.* 2012a). We found that it was similar to eukaryotic LDs in color and morphology after separating and the LDs also floated on the top of the sucrose gradient by centrifugation (Su *et al.* 2014; Zhang *et al.* 2012). Due to the lack of LD marker proteins in fish, we verified the quality of isolated LDs through several methods. Firstly, we stained purified LDs at different speeds by LipidTOX Red and confirmed the LD composition by confocal microscope. There was no other membrane contamination around the spherical LDs. Secondly, the proteins of purified LD were detected by SDS-PAGE and the result showed the proteins of liver LDs of zebrafish were significantly different from PNS, Cyto and TM. In summary, morphological and biochemical methods were used to prove that the observed spherical structure was indeed similar to the LDs of other eukaryotes and the LDs were relatively pure which was suitable for proteomic detection and analysis. Thus, the purified LDs were extracted for protein detection by mass spectrometry.

In our study, part of the identified LD-associated proteins is the same as the LD proteins from other organisms reported previously (supplementary Table S1 and Table S3). In zebrafish liver LD proteome, 259 proteins were detected and 190 proteins were reported in the proteomics of LD from other organisms. This proved the similarity between LD proteins of eukaryotes and the conservation of LD evolution since they share a large number of homologous proteins involved in lipid metabolism, membrane transport, cell signal transduction, modification protein, and proteins involved in LD interaction with various other organelles. In order to use zebrafish as a model system to study LD biology, it is important to know if the LD maker proteins exist in zebrafish. We identified the major LD maker proteins Plin2 and Plin3 and confirmed that Plin2 of zebrafish was indeed located on the LDs. Another interesting class of LD proteins shared by zebrafish and other organisms are proteins involved in membrane trafficking such as Rab10 (Beilstein *et al.* 2013; Bouchoux *et al.* 2011; Zhang *et al*. 2011). 69 proteins were unique in the zebrafish liver LD proteomic. Some proteins have been identified as lipid metabolic enzymes which are directly involved in lipid metabolism including hydroxysteroid dehydrogenase (HSD) family proteins such as Hsd17b10 and Hsdl2 that play a role in steroid metabolism (Ding *et al.* 2012b; Na *et al.* 2015; Tian *et al.* 2020; Zhang *et al.* 2012). The proteome has been identified to contain a large number of mitochondria-associated proteins such as ATP synthase and cytochrome c oxidase protein. It is unclear why the percentage of proteins involved in mitochondrial metabolism is 47% which is much higher than the ratio in mammalian LD proteomics (11%). There are two possibilities. One is that the interaction between mitochondria and LDs of zebrafish is stronger than that in mammals and most of these proteins are directly involved in the interaction. The other possibility is that the liver of zebrafish is too small and it is hard to get rid of other connective tissues completely.

Identification of new LD proteins in zebrafish is essential in studying LD dynamics. We selected five proteins from many unknown functional proteins and other proteins in the proteomics, and analyzed and determined their localization to find out whether they were LD-associated proteins in zebrafish (supplementary Fig. S1). Choline dehydrogenase and acyl carrier protein of zebrafish were located on mitochondria and retinol-binding protein 2 of zebrafish was located in cytoplasm. We also checked two uncharacterized proteins to determine if they could be located on LDs. Unfortunately, protein zgc:110339 is located in the cytoplasm, and si:ch211-113a14.11 is located in the nucleus. The results showed that new proteins targeted to LDs have not been successfully found. The reason may be that its location *in vitro* experiments did not reflect its real localization *in vivo* or the location in mammalian liver cells is different from its location in fish cells due to evolutionary reasons.

Although zebrafish has the advantage of transparent embryos, the small size of zebrafish makes it unsuitable for subsequent studies. Therefore, we want to find a model animal that is easy to obtain and the size is large enough to study. At the same time, we also want to study the proteomics of liver LDs in other fish and figure out their similarity to the proteomics of LDs in zebrafish liver.

Similar to the results of zebrafish, 267 proteins were detected in *Carassius auratus* and 146 proteins were similar to LD proteins from other organisms. Compared with other proteins, ribosome proteins were significantly upregulated in the *Carassius auratus* proteomic, which showed that more proteins play an important role in protein biosynthesis in *Carassius auratus* (Zhou *et al.* 2015). Many apolipoproteins, such as ApoA1/C1, have also been found to be involved in promoting lipid transport and regulating enzyme activity (Zhang *et al*. 2011; Westerterp *et al.* 2007), as well as a large number of other functional proteins involved in various cellular activities were detected. Similar to the proteomic of zebrafish liver, HSD and short-chain fatty acid dehydrogenase (DHS) family proteins were also identified in *Carassius auratus* proteomic. 121 proteins were newly detected in this study including ApoC2 which plays an important role in lipoprotein metabolism as an activator of lipoprotein lipase (Kei *et al.* 2012), Rdh10 which converts retinol to retinal and is required for normal embryonic development (Wu *et al.* 2002), and Pdia3, which is shown to form complexes with lectins to mediate protein folding by promoting the formation of disulfide bonds in their glycoprotein substrate (Mahmood *et al.* 2021). Compared with zebrafish proteomic, the proportion of most groups was similar. The different parts were more lipid metabolic proteins and fewer mitochondrial proteins, which may be caused by the contamination from other tissues of zebrafish due to the small size of the liver.

In conclusion, we performed the first proteomic analysis of liver LDs in zebrafish and *Carassius auratus* as model animals, and provided information on the main components of liver LD proteins. This can serve as reference data for subsequent studies on fish liver and fish LDs, using fish as model animal.

## MATERIALS AND METHODS

### Materials

HCS LipidTOX™ Red Neutral Lipid Stain and MitoTracker™ Red CMXRos were obtained from Thermo Fisher Scientific (Waltham, MA, USA). A colloidal blue staining kit was purchased from Invitrogen (Carlsbad, CA). Human Huh7 hepatocarcinoma cells were purchased from Shanghai Institutes for Biological Sciences. High glucose Dulbecco’s-Modified Eagle Medium (DMEM, C11965500BT) was purchased from Invitrogen. 10% heat-inactivated fetal bovine serum (FBS) was purchased from Invitrogen. 100 U/mL penicillin and 100 μg/mL streptomycin were purchased from Macgene Biotechnology, CN. Lipofectamine^TM^ 2000 transfection reagent was purchased from Invitrogen.

### Strains and culture conditions

Animal model zebrafish, wild type strain AB, was raised in system water at 28.5 °C under standard conditions. Zebrafish embryos were obtained by natural spawning. All zebrafish experiments were approved and carried out in accordance with the Animal Care Committee at the Institute of Zoology, Chinese Academy of Sciences, China. *Carassius auratus* were purchased from a local fish supplier.

### Isolation of LDs from zebrafish liver

LDs were isolated from zebrafish liver based on our previous method with some modifications (Ding *et al.* 2013). Briefly, the liver was carefully collected and washed in cold saline. Subsequently, the liver was transferred into 1 mL of Buffer A (25 mmol/L tricine, pH 7.8, 250 mmol/L sucrose) containing 0.2 mmol/L PMSF and homogenized with a loose-fitting glass-Teflon Dounce. Then the homogenate was centrifuged at 500 *g* for 5 min at 4 °C to collect the 500-*g* LDs. The white layer on the top was transferred into a new 1.5 mL Eppendorf tube and the supernatant was the post-nuclear supernatant (PNS). Then the PNS was transferred to a new tube for centrifugation and centrifuged at 2,000 *g* for 5 min at 4 °C to collect the 2,000-*g* LDs, and then centrifuged at 20,000 *g* for 20 min to collect 20,000-*g* LDs. After removing the LDs, the lower clear supernatant was collected as cytosol (Cyto), and the pellet was total membrane (TM). The collected LDs were then washed with 200 μL Buffer B (20 mmol/L HEPES, pH 7.4, 100 mmol/L KCl, 2 mmol/L MgCl_2_). The step that LDs were gently resuspended in 200 μL Buffer B after carefully removing the underlying solution was repeated three times. Finally, the LD proteins were precipitated with 1 mL acetone followed by a thorough vortex, and the mixture was centrifuged at 20,000 *g* for 10 min at 4 °C to drive LD proteins into a pellet. The LD proteins were subjected to proteomic analysis or were dissolved with 2× SDS sample buffer (125 mmol/L Tris Base, 20% Glycerol, 4% SDS, 4% β-mercaptoethanol and 0.04% Bromophenol blue), and denatured at 95 °C for 5 min for colloidal blue or silver staining.

### Isolation of LDs from *Carassius auratus* liver

LDs were isolated from *Carassius auratus* liver based on our previous method with some modifications (Ding *et al.* 2013). Similar to zebrafish, the liver was carefully collected and washed in cold saline. Subsequently, the liver was transferred into 10 mL Buffer A containing 0.2 mmol/L PMSF and sliced into small pieces using tweezers. After being homogenized with a loose-fitting glass-Teflon Dounce, the homogenate was centrifuged at 500 *g* for 10 min at 4 °C and the supernatant was PNS. Then PNS was transferred into a SW40 tube and Buffer B was overlaid on the top. The gradient was centrifuged at 500 *g* for 0.5 h at 4 °C. The LD layer on the top was collected carefully as 500-*g* LDs into a new tube. Then the PNS was centrifuged at 2,000 *g* for 1 h at 4 °C to collect 2,000-*g* LDs, and then centrifuged at 20,000 *g* for 1 h at 4 °C to collect 20,000-*g* LDs. After removing the LDs, the lower clear supernatant under Buffer B was collected as Cyto, and the pellet was TM. The following steps were similar to those in the purification of zebrafish LDs.

### Confocal microscopy analysis of isolated LDs

Purified LDs were resuspended with Buffer B and stained on ice for 30 min with LipidTOX Red. The ratio of LipidTOX to LD sample was 1:500 (*v*/*v*). Then, the LDs were visualized using an Olympus FV1200 Imaging System.

### Colloidal blue staining analysis

For the colloidal blue staining, the indicated proteins from different fractions were loaded on sodium dodecyl sulfate-polyacrylamide gel electrophoresis (SDS-PAGE). The fixing solution was prepared with 40 mL deionized water, 50 mL methanol and 10 mL acetic acid. The gel was put in the fixing solution and fixed for 30 min. 58 mL deionized water, 20 mL methanol, 20 mL Liquid A and 2 mL Liquid B were added into the staining solution after pouring out the fixing solution and stained for 6 h on the shaker. It can be observed after cleaning with deionized water several times.

### Silver staining analysis

For the silver staining, the indicated proteins from different fractions were loaded on SDS-PAGE. The gel was fixed in a fixing solution for 30 min and then incubated in a sensitizer solution for 30 min at room temperature. Then, the gel was washed 5 min with ddH_2_O for four times. The washed gel was treated with silver staining solution at room temperature for 20 min and followed by developing solution until bands appeared. Subsequently, the reaction was blocked by the stopping solution.

### Cell culture and transfection

Huh7 cell lines were maintained in DMEM medium supplemented with 10% FBS, 100 U/mL penicillin and 100 μg/mL streptomycin at 37 °C in a 5% CO_2_ humidified incubator.

Zebrafish *plin2* and *plin3* were cloned into the pEGFP-N1 plasmid to construct a zebrafish Plin2-GFP and Plin3-GFP fusion protein and Huh7 cells were transfected with the Plin2-GFP and Plin3-GFP plasmid using the Lipofectamine 2000 transfection reagent. Briefly, plasmids and Lipofectamine 2000 were dissolved in Opti-MEM medium for 5 min respectively. Then the plasmid solution was mixed with liposome solution gently and left for 15–20 min. The mixture of plasmid and liposome was added to Huh7 cells after aspirating the cell medium and washing twice with PBS and the medium was changed back to ordinary medium after 4–6 h. Huh7 cells were treated with or without 100 μmol/L sodium oleate (OA) for 12 h and Olympus FV1200 confocal microscopy was used to observe the cells 24 h after transfection with staining of 1:1,500 diluted LipidTOX Red and 1:1,000 diluted Hoechst for 30 min.

### Mass spectrometry and data analysis

#### Sample preparation for proteomic study on whole LD proteins and LC-MS/MS analysis

The protocol used was the same as previously described (Su *et al*. 2014). Briefly, the LD protein pellet was dissolved in 20 μL of freshly prepared 8 mol/L urea, reduced with 10 mmol/L DTT at room temperature for 1 h, and treated with 40 mmol/L iodoacetamide in the dark for 1 h to block the sulfhydryl groups. 40 mmol/L DTT was added and incubated at room temperature for 1 h to get rid of the excess IAM and diluted the sample until the urea concentration was less than 2 mol/L. Trypsin was added to a ratio of 1:50 relative to total protein content and the sample was incubated at 37 °C overnight. The digestion was terminated by adding formic acid (FA) to the final concentration of 0.5% for further detection.

The peptide mixtures were analyzed using liquid chromatography, linear ion trap and Orbitrap XL mass spectrometry (Easy nLC-LTQ-Orbitrap XL, ThermoFinnigan, San Jose, CA). The chromatographic analysis column was a C18 reverse phase column filled in the laboratory. The sample column with 150-μm id × 3 cm was filled with 5-μm ReproSil-Pur C18-AQ filler (Dr. Maisch GmbH, Ammerbuch). The analysis column with 75-μm id × 15 cm was filled with 3-μm ReproSil-Pur C18-AQ filler (Dr. Maisch GmbH, Ammerbuch). The peptides bound on the column were eluted with a 90 min linear gradient at a flow rate of 300 nL/min. Solvent A consisted of 0.1% formic acid in a water solution and Solvent B consisted of 0.1% formic acid in 80% acetonitrile solution.

Data analysis was performed by Proteome Discoverer (version 1.4.0.288, Thermo Fischer Scientific) software. The SEQUEST search engine was used to search the zebrafish or *Carassius auratus* database for the MS2 spectrogram. The search parameters were set as follows: trypsin was selected as an enzyme to digest, two missing cut sites were allowed for searching, the mass tolerance of precursor ion was set to less than 20 mg/L and the mass error of fragment ion was less than 0.6 Da. The alkylation of cysteine was specified as a fixed modification and the oxidation of methionine was chosen as a variable modification. The retrieved peptide and spectrogram matching (PSM) were filtered by the percolator algorithm with *q* values less than 1% (1% FDR). The retrieved peptides were combined into proteins using strict maximum parsimony principles.

#### Sample preparation for proteomic study on proteins in sliced gels and LC-MS/MS analysis

LD proteins were separated on a 10% SDS-PAGE gel and subjected to colloidal blue staining or silver staining. The lane with LD proteins was cut into 4 slices. In-gel digestion of each slice was performed as previously described (Ding *et al*. 2012b). Briefly, each slice was successively destained with 330 mmol/L FeK_3_(CN)_6_ and 100 mmol/L Na_2_S_2_O_3_, and then dehydrated with 100% acetonitrile. Proteins were reduced with 10 mmol/L DTT in 25 mmol/L ammonium bicarbonate at 56 °C for 1 h and alkylated by 55 mmol/L iodoacetamide in 25 mmol/L ammonium bicarbonate in the dark at room temperature for 45 min. Finally, gel pieces were thoroughly washed with 25 mmol/L ammonium bicarbonate in water-acetonitrile (1:1, *v*/*v*) solution and completely dried in a SpeedVac. Proteins were incubated for 30 min in 10 μL of modified trypsin solution (0.1 μg/μL in 25 mmol/L ammonium bicarbonate) on ice before adding 25 μL of 25 mmol/L ammonium bicarbonate and leaving overnight at 37 °C. The digestion reaction was stopped by the addition of 2% formic acid to give a final concentration of 0.1%. The gel pieces were extracted twice with fresh 80 μL 60% acetonitrile plus 0.1% formic acid, and then sonicated for 10 min. All liquid samples from the three extractions were combined and dried in a SpeedVac (ThermoFisher Scientific; Germany).

The peptide mixtures were analyzed using liquid chromatography, linear ion trap and Orbitrap XL mass spectrometry (nanoLC-LTQ-Orbitrap XL, ThermoFinnigan, San Jose, CA). The chromatographic analysis column was a C18 reverse phase column filled in the laboratory. The sample column with 150-μm id × 3 cm was filled with 5-μm ReproSil-Pur C18-AQ filler (Dr. Maisch GmbH, Ammerbuch). The analysis column with 75-μm id × 15 cm was filled with 3-μm ReproSil-Pur C18-AQ filler (Dr. Maisch GmbH, Ammerbuch). The peptides bound on the column were eluted with a 90-min linear gradient at a flow rate of 300 nL/min. Solvent A consisted of 0.5% formic acid in water solution and Solvent B consisted of 0.5% formic acid in 80% acetonitrile solution.

Data analysis was performed by Proteome Discoverer (version 1.4.0.288, Thermo Fischer Scientific) software. The SEQUEST search engine was used to search the zebrafish database for the MS2 spectrogram. The search parameters were set as follows: trypsin was selected as an enzyme to digest, two missing cut sites were allowed for searching, the mass tolerance of precursor ion was set to less than 20 mg/L and the mass error of fragment ion was less than 0.6 Da. The alkylation of cysteine was specified as a fixed modification and the oxidation of methionine was chosen as a variable modification. The retrieved peptide and spectrogram matching (PSM) were filtered by the percolator algorithm with *q* values less than 1% (1% FDR). The retrieved peptides were combined into proteins using strict maximum parsimony principles.

## Conflict of interest

Yuwei Sun, Jian Heng, Feng Liu, Shuyan Zhang and Pingsheng Liu declare that they have no conflict of interest.
